# Selective change in personal autonomy associated with motor sensory room training in children with autism: an exploratory controlled pre-post study

**DOI:** 10.3389/fpsyg.2026.1933418

**Published:** 2026-10-19

**Authors:** G. Savarese, L. Carpinelli, R. Mandia, A. Diavoletto, M. Piscitelli, F. Impemba, A. Di Siervi, A. Vertullo, F. Bottiglieri, M. Sessa, G. Corrivetti

**Affiliations:** 1 Department of Medicine and Surgery, University of Salerno, Baronissi Campus, Baronissi, Italy; 2 Cooperativa Giovamente, Salerno, Italy; 3 ASL Salerno, Salerno, Italy

**Keywords:** adaptive functioning, Autism Spectrum Disorder, exploratory controlled study, motor sensory room, pediatric neurorehabilitation, personal autonomy

## Abstract

**Background:**

Structured sensory-motor environments are increasingly used with children with Autism Spectrum Disorder (ASD), but it remains unclear whether their effects extend across multiple functional domains.

**Objective:**

This exploratory controlled pre-post study examined the pattern and specificity of behavioral and sensory change associated with a 12-week Motor Sensory Room (MSR) program.

**Methods:**

The analyzed sample comprised 54 children: 44 received MSR plus treatment as usual (TAU), and a 10-child comparison cohort received TAU alone. Ordinal behavioral items were directionally recoded into eight composites. Time x Group interactions were tested across two time points, with Bonferroni correction applied separately to the behavioral and sensory outcome families.

**Results:**

Personal autonomy was the only behavioral domain showing a correction-resistant differential improvement, *F*(1, 52) = 17.72, *p* < 0.001, Bonferroni-adjusted *p* < 0.001, partial eta squared = 0.254. No other behavioral interaction survived correction. Significant interactions were identified for four raw sensory items (rough, smooth, and soft surfaces and warm sensations); however, these primarily represented different group trajectories and could not be interpreted as clinical improvement without individual baseline hypo−/hyper-reactivity classifications.

**Conclusion:**

The findings do not support a generalized effect of MSR across behavioral or sensory functioning. They provide preliminary evidence of a selective MSR-associated change in personal autonomy that requires confirmation using randomized designs and validated adaptive-functioning measures.

## 1 Introduction

Sensory processing differences are among the core clinical characteristics of children with Autism Spectrum Disorder (ASD) and encompass a broad range of responses to environmental stimuli. According to Miller (Miller et al., 2007), these responses can be broadly categorized into three patterns: hyperreactivity, hypo-reactivity, and sensory seeking. Hyperreactivity refers to exaggerated responses to ordinary sensory stimuli, whereas hypo-reactivity involves reduced or absent responses to relevant environmental input. Sensory-seeking behaviors are characterized by repetitive actions intended to increase sensory stimulation, such as prolonged visual inspection of objects or repetitive tactile exploration.

Beyond their behavioral manifestations, sensory processing differences are increasingly recognized as reflecting atypical neural functioning. Neuroimaging and electrophysiological studies have identified altered activation in primary sensory cortices, differences in thalamocortical connectivity, and atypical multisensory integration processes in children with ASD (Marco et al., 2011; Orekhova and Stroganova, 2014; Green S. A. et al., 2015). These neurofunctional characteristics are thought to influence attention, emotional regulation, and social engagement while contributing to caregiver-reported sensory profiles (Green S. A. et al., 2015). From a clinical perspective, sensory processing differences are frequently associated with experiences of sensory overload, discomfort, or distress, which may lead to avoidance behaviors, heightened anxiety, reduced participation in everyday activities, and difficulties adapting to challenging environments (Ben-Sasson et al., 2009).

Individual variability in sensory processing can be further understood through Dunn’s sensory processing model, which conceptualizes responses according to the interaction between neurological thresholds (high versus low) and behavioral self-regulation strategies (active versus passive; Dunn, 2016). This framework describes four response patterns—sensory seeking, sensory avoiding, sensory sensitivity, and low registration—that help explain how children respond to environmental demands. Together, these complementary perspectives highlight the heterogeneity of sensory processing in ASD and underscore the need for interventions that support both sensory modulation and adaptive functioning.

Sensory processing differences across auditory, visual, vestibular, tactile, olfactory, and gustatory modalities have substantial implications for everyday functioning. They may interfere with daily living skills, personal autonomy, educational participation, and learning processes (Ashburner et al., 2008; Roley et al., 2009; Whitby and Mancil, 2009; Keen et al., 2016; Thye et al., 2018). Consistent with this evidence, atypical sensory responses are included among the diagnostic characteristics of ASD in the DSM-5 (American Psychiatric Association, 2013), with prevalence estimates ranging from 69 to 93% during childhood and adolescence. Emerging evidence also suggests that sensory processing patterns may have a familial component, as atypical sensory profiles have been reported among parents of children with ASD (Uljarević et al., 2016; Donaldson et al., 2017; Glod et al., 2017). Although the developmental trajectory of sensory processing differences is heterogeneous, sensory over-responsivity (SOR) often emerges early in childhood (Green J. et al., 2015; Perez Repetto et al., 2017) and has been associated with greater autistic traits during school age (Chen et al., 2022), higher levels of anxiety, and increased internalizing and externalizing behaviors (Green et al., 2017). While some longitudinal studies suggest partial improvement over time (Carson et al., 2024), sensory processing differences frequently continue to affect daily functioning and contribute to increased caregiver stress (Jasmin et al., 2009). Their association with adaptive functioning appears to vary according to age, cognitive level, and developmental domain (Kaur et al., 2018; Loh et al., 2023).

Motor difficulties are also highly prevalent among children with ASD, affecting approximately 50–85% of individuals (Wiggins et al., 2024). Common manifestations include impairments in gross and fine motor skills, coordination, motor planning, postural control, gait, perceptual–motor integration, and manual dexterity (Coll et al., 2020; Wang et al., 2022). Both the DSM-5 (American Psychiatric Association, 2013) and ICD-11 (World Health Organization, 2019) recognize that children with ASD may also meet diagnostic criteria for Developmental Coordination Disorder (DCD), although the mechanisms underlying motor impairments may only partially overlap between these conditions (Miller et al., 2024). Given the close interaction between sensory processing and motor performance, interventions targeting both domains have received increasing attention.

In recent years, structured sensory–motor environments have been increasingly adopted to provide controlled multisensory experiences integrating visual, auditory, tactile, vestibular, proprioceptive, olfactory, and gustatory stimulation. Preliminary evidence suggests that these environments may facilitate sensory regulation, engagement, social participation, and adaptive functioning while reducing extreme patterns of sensory hyper- and hypo-reactivity (De Domenico et al., 2024; Awaida et al., 2024). Nevertheless, the available literature remains methodologically heterogeneous. Most published studies are characterized by small samples, uncontrolled designs, or limited descriptions of intervention protocols, making it difficult to determine the specific contribution of sensory–motor training beyond the effects of usual care and limiting the reproducibility of findings across clinical settings.

The Motor Sensory Room approach examined in the present study was developed within this emerging field and is conceptually grounded in the interaction between sensory modulation, motor organization, arousal regulation, and adaptive behavioral functioning. The intervention combines graded multisensory experiences with active motor engagement to support self-regulation, participation, and adaptive responses within a structured therapeutic environment. Although informed by key principles of Ayres Sensory Integration (ASI), including child-directed engagement, multisensory input, and the promotion of adaptive motor responses, the intervention was not implemented according to the standardized ASI Fidelity Measure and should therefore be considered an ASI-informed rather than an ASI intervention (Schaaf et al., 2014; Alvarez-Fernandez et al., 2017; Schoen et al., 2019).

This conceptual framework is consistent with an expanding body of literature supporting the use of structured sensory-motor interventions for children with ASD (Hill et al., 2012; Essary et al., 2020; Spitale et al., 2020; Park et al., 2021). Furthermore, a preliminary observational study conducted by our research group (Savarese et al., 2025) described improvements in sensory regulation and motor coordination following Motor Sensory Room training, providing the rationale for further investigation using a more rigorous study design.

Despite increasing clinical interest, evidence regarding structured sensory-motor interventions remains limited by small samples, uncontrolled or quasi-experimental designs, heterogeneous protocols, and inconsistent outcome measurement. Few studies have compared sensory-motor training with treatment as usual while assessing several potentially responsive domains within the same design. Such multi-domain evaluation is useful not only for identifying possible benefits, but also for determining whether change is broad or restricted to particular functional outcomes.

Accordingly, this exploratory controlled pre-post study compared children receiving Motor Sensory Room training in addition to usual care with a comparison group receiving treatment as usual alone. Rather than assuming a uniform intervention effect, we examined the pattern and specificity of differential change across sensory processing, motor functioning, adaptive behavior, and socio-communicative functioning. The prespecified domain-level hypotheses were that MSR participants would show: (H1) greater reductions in sensory hyper- and hypo-reactivity; (H2) greater improvement in motor coordination and balance; (H3) greater gains in adaptive daily-living skills; and (H4) greater improvement in socio-communicative functioning. Given the number and heterogeneity of outcomes, family-wise multiplicity correction was used, and each hypothesis was considered supported only when the corresponding controlled interaction remained significant after correction.

## 2 Materials and methods

### 2.1 Study design and participants

This study employed a controlled pre–post quasi-experimental design to evaluate the effects of a structured Motor Sensory Room intervention in children with Autism Spectrum Disorder (ASD). Participants were recruited consecutively from children referred to the Child Neuropsychiatry Unit of the Local Health Authority (ASL Salerno, Italy) for the diagnostic assessment of neurodevelopmental disorders.

During the recruitment period, 51 children with a confirmed ASD diagnosis were screened for inclusion in the MSR intervention group. Four did not complete the post-intervention assessment and three were excluded because of comorbid neurological conditions. Accordingly, the complete-case MSR analysis included 44 children who attended at least 80% of scheduled sessions and completed both T1 and T2. The separately identified TAU cohort comprised 10 additional children and was not included in the figure of 51 screened for MSR participation. Thus, the total analyzed sample comprised 54 children (44 MSR and 10 TAU).

The comparison group comprised 10 children with ASD who received treatment as usual (TAU) during the same study period and were not exposed to the Motor Sensory Room intervention. They continued their standard educational and therapeutic programs and underwent the same assessment schedule at baseline and post-intervention.

ASD diagnoses were established by multidisciplinary teams including child neuropsychiatrists and clinical psychologists according to the DSM-5 criteria (American Psychiatric Association, 2013). Severity levels (Levels 1–3) were assigned following DSM-5 recommendations.

Eligibility criteria included: (a) a diagnosis of ASD; (b) age between 2 and 11 years; (c) completion of both assessment sessions; and (d) attendance at 80% or more of the scheduled intervention sessions for participants allocated to the intervention group. Exclusion criteria were: (a) neurological or genetic disorders likely to affect neurodevelopment (e.g., epilepsy, Rett syndrome, or known chromosomal abnormalities); (b) severe sensory or motor impairments preventing participation in the intervention; and (c) major changes in pharmacological treatment during the study.

The intervention group comprised 44 children with complete pre-post data. At the demographic reference date of 1 January 2025, their mean age was 6.59 years (SD = 2.27; range = 2.12–10.77); 38 were boys and 6 were girls. Four were classified as DSM-5 Level 1, 16 as Level 2, and 22 as Level 3; severity data were unavailable for two participants. Fourteen had a history of preterm birth. Nineteen were nonverbal, 13 minimally verbal, and 12 verbal.

Throughout the study, all participants continued to receive their usual educational and therapeutic support, including psychoeducational interventions, speech and language therapy, behavioral programs, and school-based support when indicated. None of the children enrolled in the intervention group had previously participated in structured sensory–motor or Ayres Sensory Integration (ASI)-based programs. No clinically relevant changes in pharmacological treatment occurred during the study period. Nevertheless, the potential influence of concurrent interventions and developmental maturation cannot be completely excluded (Table 1).

**Table 1:** Baseline characteristics of the MSR and TAU groups.

Baseline characteristic	MSR (*n* = 44)	TAU (*n* = 10)
Age at 1 January 2025, M ± SD (range)	6.59 ± 2.27 (2.12–10.77)	6.68 ± 1.85 (4.01–9.29)
Sex, n (%)	38 male (86.4%); 6 female (13.6%)	8 male (80.0%); 2 female (20.0%)
ASD severity, n (%)	Level 1: 4 (9.1%); Level 2: 16 (36.4%); Level 3: 22 (50.0%); missing: 2 (4.5%)	Level 1: 2 (20.0%); Level 2: 2 (20.0%); Level 3: 6 (60.0%)
Verbal status, n (%)	Nonverbal: 19 (43.2%); minimally verbal: 13 (29.5%); verbal: 12 (27.3%)	Nonverbal: 5 (50.0%); minimally verbal: 2 (20.0%); verbal: 3 (30.0%)
Prematurity, n (%)	Preterm: 14 (31.8%); full-term: 30 (68.2%)	Preterm: 3 (30.0%); full-term: 7 (70.0%)

Absolute SMDs based on the displayed data were 0.04 for age, 0.17 for sex, 0.31 for ASD Level 1, 0.37 for ASD Level 2, 0.20 for ASD Level 3, 0.14 for nonverbal status, 0.22 for minimally verbal status, 0.06 for verbal status, and 0.04 for prematurity. Severity percentages use the full MSR denominator (*n* = 44), including two missing observations.

### 2.2 Measures

A structured sociodemographic and clinical interview was administered to parents and integrated with direct clinical observation to characterize feeding, sleep, coordination, play, personal autonomy, communication, social interaction, and behavioral regulation. The 35 study-specific behavioral items and 13 sensory-stimulus items were coded on four-point ordinal scales (0 = none, 1 = low, 2 = medium, 3 = high). For positively framed ability items, higher scores indicated more consistent or independent functioning; difficulty items were reverse-scored only when behavioral composites were calculated. Raw sensory scores represented the intensity and functional impact of the observed response and were not interpreted as improvement without reference to the child’s baseline hypo- or hyper-reactivity profile. The complete item list, scoring direction, operational anchors, and coding rules are provided in Supplementary material S1. These study-specific variables were used for exploratory outcome assessment and were not treated as standardized PEP-3 or Sensory Profile-2 subscales.

Two observers were present during all assessments, and a score was entered only when both observers agreed on the category. Agreement was reached for every recorded rating. Only the single agreed score was retained in the study database; separate observer-level ratings and pre-consensus ratings were not archived. Consequently, a chance-corrected inter-rater coefficient (e.g., weighted kappa or ICC) cannot be reconstructed retrospectively. This consensus-coding procedure represents a methodological limitation; future studies should retain independent ratings for formal reliability analysis.

Cognitive, communicative, motor, and socio-emotional functioning were assessed using the Italian version of the Psychoeducational Profile-Third Edition (PEP-3; Schopler et al., 2005), a standardized instrument specifically designed for individuals with ASD. The PEP-3 provides performance-based measures across developmental domains, including communication (expressive and receptive), motor skills (fine and gross), and cognitive abilities, as well as observational scales assessing maladaptive behaviors and relational patterns. The instrument yields developmental profiles used to identify individual strengths and areas of difficulty. The Italian adaptation demonstrates good psychometric properties, with satisfactory internal consistency and test–retest reliability. Administration was conducted individually by trained clinicians in a standardized clinical setting.

Sensory responses were evaluated using program-specific ordinal observations integrated with the PEP-3 assessment framework, as described in the preliminary report of this cohort (Savarese et al., 2025). Responses to long, short, and loud sounds; lights and video projection; solid and liquid foods; rough, smooth, and soft surfaces; cold and warm sensations; and strong smells were coded as none (0), low (1), medium (2), or high (3).

All assessments were administered at two time points: baseline (T1), prior to the intervention, and post-intervention (T2), immediately following the 12-week training period.

### 2.3 Motor sensory room intervention

Children allocated to the experimental group participated in a structured Motor Sensory Room (MSR) program delivered over 12 consecutive weeks. The Motor Sensory Room is a standardized multisensory environment developed by the Cooperative Giovamente (Salerno, Italy) to promote sensory modulation, motor organization, emotional regulation, and adaptive functioning through individualized sensory–motor experiences. The intervention was delivered according to the standardized Motor Sensory Room operational protocol adopted across participating centers.

Participants attended two individual sessions per week, each lasting approximately 60 min, for a total of 24 sessions. All sessions were conducted by therapists specifically trained in developmental and sensory–motor interventions and followed the same predefined sequence of activities (see Table 2). Although therapeutic tasks were individualized according to each child’s sensory profile and behavioral responses, the temporal organization, therapeutic objectives, and progression of each session were standardized.

**Table 2:** Standardized motor sensory room intervention protocol.

Phase	Time (min)	Therapeutic objective	Main activities	Expected therapeutic outcome
Welcome and regulation	10	Facilitate transition and emotional regulation	Greeting routine, breathing, calming music	Reduced arousal and therapeutic engagement
Free sensory exploration	10	Identify sensory preferences and spontaneous regulation	Child-led exploration of sensory equipment	Increased engagement and therapist observation
Guided sensory–motor activities	15	Promote sensory integration, balance, coordination and motor planning	Obstacle course, tactile, vestibular and proprioceptive activities	Improved sensory modulation and adaptive responses
Pre-heated sensory immersion	15	Promote deep-pressure stimulation, proprioception and relaxation	Immersion in temperature-controlled sensory pool	Enhanced body awareness, emotional regulation and self-regulation
Reorientation	5	Return to baseline arousal	Gradual reduction of sensory input and verbal/non-verbal processing	Smooth transition to the external environment
Therapist documentation	5	Monitor intervention fidelity and individual progress	Structured observational checklist	Treatment monitoring and individualized planning

Each session consisted of six consecutive phases. During the initial welcoming and regulation phase (approximately 10 min), the therapist facilitated the transition into the multisensory environment through calming routines, such as breathing exercises, soft music, or brief relational rituals, to promote emotional regulation and readiness for participation. This was followed by a period of free exploration (approximately 10 min), during which children were encouraged to interact spontaneously with the sensory equipment while the therapist adopted a primarily observational role, identifying preferences, sensory responses, and emerging regulatory needs.

The guided sensory–motor phase (approximately 15 min) involved individualized activities targeting sensory integration and motor organization, including obstacle courses, tactile exploration, balance exercises, visual tracking, proprioceptive tasks, and graded vestibular stimulation. Activities were selected according to each child’s sensory profile while maintaining common therapeutic objectives across participants.

A distinctive component of the intervention was the pre-heated sensory immersion pool, in which children spent approximately 15 min experiencing deep-pressure stimulation within a thermally regulated environment containing soft sensory materials. During this phase, therapists adopted a non-directive, co-regulatory role, providing emotional containment while allowing children to self-regulate their level of interaction with the environment. This component was designed to promote body awareness, proprioceptive processing, relaxation, and emotional regulation.

The intervention concluded with a reorientation phase (approximately 5 min), during which sensory stimulation was gradually reduced, and children were supported in transitioning back to their everyday environment through verbal, symbolic, or non-verbal processing of the experience. Immediately after each session, therapists completed a structured observational record documenting behavioral responses, emotional regulation, sensory modulation, engagement, and recommendations for subsequent sessions. These records supported ongoing individualization while ensuring consistency across treatment sessions.

The Motor Sensory Room integrates controlled visual, auditory, tactile, vestibular, proprioceptive, olfactory, and gustatory stimulation within a protected therapeutic environment. Throughout the program, therapists continuously adjusted the intensity and complexity of activities according to children’s behavioral regulation and sensory tolerance while preserving the standardized structure of the protocol. Intervention fidelity was supported through therapist training, the use of standardized operational procedures, structured session documentation, and regular clinical supervision. Although conceptually informed by key principles of Ayres Sensory Integration (ASI), including active participation, multisensory engagement, and adaptive motor responding, the program was not delivered according to the ASI Fidelity Measure and should therefore be considered an ASI-informed sensory–motor intervention rather than a standardized ASI protocol (Schaaf et al., 2014; Alvarez-Fernandez et al., 2017; Schoen et al., 2019).

### 2.4 Study procedure

Participants were not randomized. The MSR group comprised 44 children with complete pre-post data who accessed the 12-week program. The TAU group consisted of the same 10-child comparison cohort described in the preliminary study (Savarese et al., 2025). The children were drawn from the same clinical context and selected for similarity in age, sex, and ASD diagnosis/severity. They continued ABA-based rehabilitation, psychomotor intervention, and speech therapy without MSR exposure. Because allocation and selection were non-random, selection bias and residual confounding remain possible.

At baseline (T1), all participants underwent the same assessment protocol, including the structured sociodemographic and clinical interview, the PEP-3, and the program-specific sensory-response observations described above. Assessments were conducted in a controlled clinical setting by an experienced clinician independent of the intervention team and blinded to group allocation whenever possible.

Following baseline assessment, children in the intervention group participated in the 12-week Motor Sensory Room program while continuing to receive their usual educational and therapeutic interventions. The comparison group continued to receive treatment as usual over the same time period and did not receive any structured sensory–motor intervention.

At the end of the 12-week period (T2), all participants completed the same assessment battery administered at baseline. The interval between T1 and T2 was equivalent across groups to ensure temporal comparability.

Treatment adherence in the intervention group was monitored throughout the study. Inclusion in the final analyses required attendance to at least 80% of scheduled Motor Sensory Room sessions. In addition, therapists completed structured observational records after each session to document behavioral responses, sensory modulation, emotional regulation, and engagement. These records were used exclusively for clinical monitoring and intervention fidelity and were not included in statistical analyses.

### 2.5 Statistical analysis

Ordinal categories were coded as 0 = none, 1 = low, 2 = medium, and 3 = high. For behavioral composites, negatively worded items were reverse-scored so that higher values consistently indicated better functioning; item scores were averaged within feeding, sleep, coordination, play, personal autonomy, communication, social interaction, and behavioral regulation. Sensory items were analyzed in their raw ordinal direction because clinical meaning depends on baseline hypo−/hyper-reactivity. With two time points, the Time × Group interaction was evaluated by comparing individual change scores between MSR and TAU; the resulting interaction F statistic is the square of the equal-variance independent-samples t statistic and has df = 1.52. Mann–Whitney tests of change scores were used as ordinal/small-sample sensitivity analyses.

Baseline characteristics of the Motor Sensory Room (MSR) and treatment-as-usual (TAU) groups were summarized using means, standard deviations, ranges, counts, and percentages. Absolute standardized mean differences (SMDs) were calculated descriptively for continuous and categorical characteristics. Because the TAU group was small and allocation was non-random, baseline equivalence was not inferred from significance testing.

Intervention effects were examined using Time (pre-intervention vs. post-intervention) as the within-subject factor and Group (MSR vs. TAU) as the between-subject factor. The Time x Group interaction was the primary indicator of differential change between groups and was not interpreted as proof of causal efficacy because allocation was non-random.

Separate analyses were conducted for each of the eight behavioral composites and each of the 13 raw sensory-stimulus items. The sensory variables were study-specific observational items rather than standardized Sensory Profile-2 subscales.

Effect sizes were expressed as partial eta squared (η^2^p), with 95% confidence intervals reported for between-group differences in change. Family-wise Bonferroni correction was applied separately across eight behavioral domains and 13 sensory items. Thus, adjusted *p* values equal min(8p, 1) for behavioral outcomes and min(13p, 1) for sensory outcomes. Two-tailed α was 0.05.

Given the developmental heterogeneity of the sample, an exploratory analysis was conducted including Age Group (preschool vs. school-age) as an additional between-subject factor to examine potential moderating effects on treatment response.

All statistical tests were two-tailed, with a significance threshold set at *p* < 0.05. Analyses were performed using standard statistical software IBM SPSS.

### 2.6 Ethical considerations

The study was conducted in accordance with the Declaration of Helsinki and approved by the Independent Ethics Committee of the Psychological Counseling Center of the University of Salerno (UNISA; approval number 1, dated 12 July 2021). All procedures involving human participants were carried out in accordance with institutional and national ethical standards.

The present extended analysis and the preliminary report derived from the same research protocol, cohort framework, MSR procedures, and data collection covered by approval no. 1 of 12 July 2021.

Written informed consent was obtained from the parents or legal guardians of all participants prior to enrolment in the study. Assent was also obtained from children whenever developmentally appropriate.

Participation was voluntary, and families were informed that they could withdraw from the study at any time without any consequences for ongoing clinical care.

All data were anonymized prior to analysis to ensure confidentiality and privacy protection. The study adhered to relevant data protection regulations and ethical guidelines for research involving minors and clinical populations.

Although the study design followed a quasi-experimental controlled pre–post approach, reporting was aligned with STROBE guidelines for observational studies.

## 3 Results

### 3.1 Sample characteristics

The total analysed sample comprised 54 children: 44 in the MSR group and 10 in the TAU group. The MSR sample included 38 boys and 6 girls. At 1 January 2025, their mean age was 6.59 years (SD = 2.27; range = 2.12–10.77). Fourteen children (31.8%) were born preterm. ASD severity was Level 1 for 4 children (9.1%), Level 2 for 16 (36.4%), and Level 3 for 22 (50.0%); severity data were unavailable for 2 children (4.5%). Nineteen children (43.2%) were nonverbal, 13 (29.5%) minimally verbal, and 12 (27.3%) verbal.

With respect to clinical severity in the MSR group, 4 children (9.1%) were classified as ASD Level 1, 16 (36.4%) as Level 2, and 22 (50.0%) as Level 3 according to DSM-5 criteria; severity data were unavailable for 2 children (4.5%).

From a communicative standpoint in the MSR group, 19 children (43.2%) were nonverbal, 13 (29.5%) minimally verbal, and 12 (27.3%) demonstrated functional verbal language.

Baseline adaptive functioning indicated marked difficulties in basic self-care domains: toilet training had not yet been acquired in 56.8% of the sample, personal hygiene was reported as inadequate in 59.1%, and food selectivity was present in 59.1% of participants.

Relational difficulties were frequently reported both in peer interactions—rated as mild in 18.2%, moderate in 47.7%, and severe in 34.1% of cases—and in interactions with adults (15.9% mild, 40.9% moderate, 43.2% severe). Reactions to unpredictable situations were predominantly severe (61.4%), as were responses in non-protected or unfamiliar contexts (59.1%). Provocative or oppositional behaviors were described as low in 50% of participants, moderate in 25%, and high in 25% (Table 3).

**Table 3:** Baseline demographic and clinical characteristics of the MSR group (*N* = 44).

Variable	Value
Sex	Male: 38 (86.4%); Female: 6 (13.6%)
ASD severity (DSM-5)	Level 1: 4 (9.1%); Level 2: 16 (36.4%); Level 3: 22 (50.0%); Missing: 2 (4.5%)
Verbal status	Nonverbal: 19 (43.2%); Minimally verbal: 13 (29.5%); Verbal: 12 (27.3%)
Prematurity	Preterm: 14 (31.8%); Full-term: 30 (68.2%)

Regarding sensory processing patterns at baseline, descriptive profiles indicated a higher prevalence of hyper-reactivity in the visual (54.5%), auditory (65.9%), and tactile (65.9%) modalities, whereas hypo-reactivity was more frequently observed in the olfactory (59.1%) and gustatory (52.3%) domains.

### 3.2 Pre–post intervention comparison: behavioral domains

Table 4 presents descriptive MSR-group composite scores at T1 and T2. All eight domain means increased after directional recoding (higher scores = better functioning). These within-group descriptives are not used to establish efficacy; controlled Time × Group tests are reported in Section 3.5.

**Table 4:** Pre-post comparison of behavioral domains in the MSR group (*N* = 44).

Domain	MSR T1 (M ± SD)	MSR T2 (M ± SD)	Mean change
Feeding	1.66 ± 0.47	1.88 ± 0.49	0.21
Sleep	1.44 ± 0.78	1.88 ± 0.63	0.44
Coordination	1.73 ± 0.50	2.16 ± 0.43	0.44
Play	0.78 ± 0.47	1.22 ± 0.45	0.44
Personal autonomy	0.44 ± 0.45	0.86 ± 0.47	0.42
Communication	1.33 ± 0.73	1.62 ± 0.82	0.29
Social interaction	1.03 ± 0.50	1.52 ± 0.51	0.49
Behavioral regulation	1.46 ± 0.64	1.91 ± 0.48	0.45

### 3.3 Pre–post intervention comparison: observed sensory reactions

Table 5 presents descriptive MSR-group raw sensory-item scores at T1 and T2. Because score direction depends on baseline hypo−/hyper-reactivity, numerical change is not labeled improvement. Controlled Time × Group tests are reported in Section 3.6.

**Table 5:** Pre-post comparison of observed sensory reactions in the MSR group (*N* = 44).

Modality	Stimulus item	MSR T1 (M ± SD)	MSR T2 (M ± SD)
Auditory	Long-duration sound	2.02 ± 0.85	1.93 ± 0.50
Auditory	Short-duration sound	1.34 ± 0.91	1.61 ± 0.58
Auditory	Loud sound	2.59 ± 0.62	2.27 ± 0.59
Visual	Lights	2.25 ± 0.69	2.14 ± 0.46
Visual	Video projection	2.32 ± 0.71	2.05 ± 0.53
Gustatory	Solid food	2.36 ± 0.81	2.14 ± 0.63
Gustatory	Liquid food	1.66 ± 0.57	1.75 ± 0.53
Tactile	Rough surfaces	2.07 ± 0.70	2.02 ± 0.59
Tactile	Smooth surfaces	1.82 ± 0.58	1.95 ± 0.43
Tactile	Soft surfaces	2.11 ± 0.65	2.09 ± 0.42
Tactile	Cold sensations	2.18 ± 0.92	2.14 ± 0.59
Tactile	Warm sensations	1.91 ± 0.68	1.93 ± 0.45
Olfactory	Withdrawal from strong odors	1.30 ± 1.09	1.55 ± 0.79

### 3.4 Baseline comparability between groups (MSR vs. TAU)

Baseline characteristics for both groups are presented in Table 1. Absolute SMDs were small for age and prematurity (0.04 each), but larger for sex (0.17), verbal-status categories (0.06–0.22), and ASD-severity categories (0.20–0.37). These imbalances, together with the small non-random TAU cohort, preclude any claim of baseline equivalence and must be considered when interpreting the controlled comparisons.

### 3.5 Intervention effects on behavioral functioning (time × group interactions)

Complete behavioral results are shown in Table 6. After Bonferroni correction across eight domains, only personal autonomy showed a significant Time × Group interaction, *F*(1,52) = 17.72, *p* = 0.000102, adjusted *p* = 0.000812, η^2^p = 0.254. Personal autonomy increased in the MSR group from M = 0.44 (SD = 0.45) to M = 0.86 (SD = 0.47), while the TAU group remained unchanged at M = 0.77 (SD = 0.59). The between-group difference in change was 0.42 points (95% CI [0.22, 0.63]); the Mann–Whitney sensitivity test was also significant (*p* < 0.001). No other behavioral interaction remained significant after correction.

**Table 6:** Behavioral outcomes by group and time with complete Time x Group interaction statistics.

Outcome	MSR T1	MSR T2	TAU T1	TAU T2	F(df1,df2)	p / adjusted p	η^2^p; Δ 95% CI
Feeding	1.66 ± 0.47	1.88 ± 0.49	1.68 ± 0.33	1.65 ± 0.36	5.02 (1,52)	0.029420/0.235357	0.088; Δ CI [0.02, 0.45]
Sleep	1.44 ± 0.78	1.88 ± 0.63	1.88 ± 0.65	2.17 ± 0.49	2.42 (1,52)	0.125528/1.000000	0.045; Δ CI [−0.04, 0.33]
Coordination	1.73 ± 0.50	2.16 ± 0.43	1.93 ± 0.29	2.18 ± 0.30	4.27 (1,52)	0.043681/0.349452	0.076; Δ CI [0.01, 0.37]
Play	0.78 ± 0.47	1.22 ± 0.45	1.14 ± 0.34	1.46 ± 0.44	2.69 (1,52)	0.106995/0.855959	0.049; Δ CI [−0.03, 0.27]
Personal autonomy	0.44 ± 0.45	0.86 ± 0.47	0.77 ± 0.59	0.77 ± 0.59	17.72 (1,52)	0.000102/0.000812	0.254; Δ CI [0.22, 0.63]
Communication	1.33 ± 0.73	1.62 ± 0.82	1.60 ± 0.50	1.73 ± 0.46	2.77 (1,52)	0.101910/0.815280	0.051; Δ CI [−0.03, 0.36]
Social interaction	1.03 ± 0.50	1.52 ± 0.51	1.25 ± 0.26	1.65 ± 0.41	0.88 (1,52)	0.351692/1.000000	0.017; Δ CI [−0.10, 0.28]
Behavioral regulation	1.46 ± 0.64	1.91 ± 0.48	1.50 ± 0.67	1.82 ± 0.64	0.97 (1,52)	0.329369/1.000000	0.018; Δ CI [−0.13, 0.39]

Unadjusted and Bonferroni-adjusted *p* values are reported.

### 3.6 Intervention effects on sensory processing (time × group interactions)

Complete sensory results are shown in Table 7. After correction across 13 items, significant Time × Group interactions were observed for responses to rough surfaces, *F*(1,52) = 18.14, adjusted *p* = 0.0011, η^2^p = 0.259; smooth surfaces, *F*(1,52) = 14.69, adjusted *p* = 0.0045, η^2^p = 0.220; soft surfaces, *F*(1,52) = 14.86, adjusted *p* = 0.0042, η^2^p = 0.222; and warm sensations, *F*(1,52) = 14.44, adjusted *p* = 0.0049, η^2^p = 0.217. These interactions mainly reflected marked score reductions in TAU and relative stability in MSR. Because the raw ordinal score does not have a uniform improvement direction across children with hypo- versus hyper-reactivity, these findings demonstrate different trajectories but cannot be labeled clinical improvements. No auditory, visual, gustatory, olfactory, or cold-sensation item survived Bonferroni correction.

**Table 7:** Sensory-item descriptives and Bonferroni-adjusted Time × Group interactions.

Sensory item	MSR T1	MSR T2	TAU T1	TAU T2	*F*(1,52)	Adjusted p	η^2^p
Long-duration sound	2.02 ± 0.85	1.93 ± 0.50	2.40 ± 0.52	2.10 ± 0.74	0.72	1.0000	0.014
Short-duration sound	1.34 ± 0.91	1.61 ± 0.58	2.30 ± 0.82	2.00 ± 0.94	7.38	0.1160	0.124
Loud sound	2.59 ± 0.62	2.27 ± 0.59	2.70 ± 0.48	2.10 ± 0.74	2.11	1.0000	0.039
Lights	2.25 ± 0.69	2.14 ± 0.46	1.90 ± 0.57	1.50 ± 0.53	2.34	1.0000	0.043
Video projection	2.32 ± 0.71	2.05 ± 0.53	1.80 ± 0.92	1.50 ± 0.97	0.02	1.0000	0.000
Solid food	2.36 ± 0.81	2.14 ± 0.63	2.20 ± 0.92	2.20 ± 0.92	1.87	1.0000	0.035
Liquid food	1.66 ± 0.57	1.75 ± 0.53	1.80 ± 0.63	1.80 ± 0.63	0.36	1.0000	0.007
Rough surfaces	2.07 ± 0.70	2.02 ± 0.59	2.50 ± 0.53	1.80 ± 0.42	18.14	0.0011	0.259
Smooth surfaces	1.82 ± 0.58	1.95 ± 0.43	2.10 ± 0.74	1.60 ± 0.70	14.69	0.0045	0.220
Soft surfaces	2.11 ± 0.65	2.09 ± 0.42	2.60 ± 0.52	1.90 ± 0.57	14.86	0.0042	0.222
Cold sensations	2.18 ± 0.92	2.14 ± 0.59	2.00 ± 1.05	1.40 ± 0.97	6.42	0.1868	0.110
Warm sensations	1.91 ± 0.68	1.93 ± 0.45	1.80 ± 1.03	1.20 ± 0.92	14.44	0.0049	0.217
Withdrawal from strong odors	1.30 ± 1.09	1.55 ± 0.79	1.00 ± 0.67	0.70 ± 0.67	4.52	0.4972	0.080

Raw 0–3 scores; clinical direction depends on baseline sensory profile.

All sensory-item descriptives and interaction statistics are reported in Table 7.

### 3.7 Exploratory analysis: age-related effects on intervention response

The Time × Group × Age Group analyses were exploratory and no statistically significant three-way interactions were identified. Given the very small subgroup sizes and limited power, descriptive differences by age are considered hypothesis-generating only; non-significance is not evidence that age does not moderate response.

Given the small size of the preschool subgroup, these findings should be interpreted cautiously and considered hypothesis-generating.

## 4 Discussion

This exploratory controlled study examined whether change associated with Motor Sensory Room participation was generalized across multiple outcomes or selective to particular functional domains.

The principal finding was selective rather than global: after comparison with TAU and correction for multiplicity, personal autonomy was the only behavioral domain demonstrating a robust differential change. Given the quasi-experimental design, this result indicates an association with MSR participation and does not establish causality.

The effect for personal autonomy was substantial (partial eta squared = 0.254), and the between-group difference in change was supported by both the parametric interaction and the Mann–Whitney sensitivity analysis. In contrast, the remaining behavioral domains did not survive family-wise correction. The evidence therefore supports a narrow adaptive-functioning signal, not a general improvement in behavior, communication, motor coordination, feeding, sleep, play, or social interaction.

Considered against the original hypotheses, H3 received partial support limited to personal autonomy. H2 and H4 were not supported because motor coordination and socio-communicative outcomes did not show correction-resistant differential improvement. H1 was not clinically confirmed: although four tactile/temperature interactions survived correction, their direction could not be classified as beneficial from the raw ordinal scores.

The selective autonomy finding is compatible with the possibility that a structured and repeatedly practiced therapeutic environment may first influence routine participation, initiation, and supported independence. This interpretation remains provisional because autonomy was measured with a study-specific composite and potential mechanisms, including arousal regulation or transfer of therapist-supported routines to everyday contexts, were not directly assessed.

Personal autonomy is nevertheless a clinically relevant outcome because it concerns participation in everyday routines and dependence on caregivers. The size and consistency of the observed interaction justify focused replication with validated measures such as standardized adaptive-behavior scales and with item-level information capable of distinguishing dressing, washing, initiation, and need for adult prompting.

No controlled evidence remained for improvements in social interaction or behavioral regulation after multiplicity correction. Their descriptive pre-post changes must therefore not be interpreted as intervention effects. These null findings are informative because they suggest that a 12-week MSR program, as delivered here, should not be expected to produce broad short-term change across complex socio-communicative and regulatory domains.

Likewise, motor coordination, communication, feeding, sleep, and play did not demonstrate correction-resistant differential improvement. These functions may require more targeted or longer interventions, but the present data cannot distinguish insufficient treatment intensity from limited measurement sensitivity, developmental heterogeneity, concurrent treatment effects, or true absence of benefit.

Four tactile/temperature sensory items showed statistically different trajectories between groups. However, the raw 0–3 coding quantified response magnitude without identifying whether a particular child was hypo-reactive or hyper-reactive at baseline. Moreover, the interactions mainly reflected reductions in TAU and relative stability in MSR. These results should therefore be treated as exploratory trajectory differences rather than evidence of sensory improvement.

The contribution of the study is therefore one of specificity: a potentially meaningful signal emerged for personal autonomy, whereas generalized behavioral and sensory benefits were not demonstrated. Transparent reporting of this pattern avoids overstatement and provides a more precise basis for designing confirmatory studies with a prespecified autonomy endpoint.

## 5 Conclusion

This exploratory controlled reanalysis provides preliminary evidence of a selective MSR-associated improvement in personal autonomy. It does not support generalized efficacy across behavioral, motor, socio-communicative, or sensory outcomes.

After comparison with TAU and correction for multiplicity, personal autonomy was the only behavioral domain showing a robust differential change. Accordingly, the adaptive-functioning hypothesis was only partially supported, while the motor and socio-communicative hypotheses were not supported.

The significant tactile/temperature interactions indicate different group trajectories but cannot be interpreted as clinical sensory improvement from the available raw scores. The sensory hypothesis therefore remains unconfirmed.

Interpretation is limited by the quasi-experimental design, retrospective selection and very small TAU group, probable selection bias and residual confounding, absence of an *a priori* power calculation, unbalanced groups, multiple comparisons, a study-specific and incompletely validated autonomy composite, absence of standardized fidelity measurement, broad age range, and concurrent interventions. The autonomy result should therefore be considered hypothesis-generating and confirmed in a randomized, adequately powered study using a validated adaptive-behavior instrument and a prespecified primary endpoint.

An important limitation concerns the broad age range of the participants, spanning from early preschool years to late childhood. Sensory processing, motor abilities, adaptive functioning, and socio-communicative skills undergo substantial developmental changes across this age period. Consequently, children of different ages may present distinct baseline profiles and may respond differently to sensory–motor interventions. Although an exploratory analysis including age group as a moderating factor did not reveal statistically significant effects, the relatively small sample size, particularly within the preschool subgroup, limited the statistical power to detect developmental differences. Therefore, the observed intervention effects should be interpreted as averaged across a heterogeneous developmental population. Future studies should recruit larger age-specific samples and examine intervention outcomes separately in preschool- and school-aged children to better understand potential developmental variations in treatment.

The present study extends the previously published preliminary report (Savarese et al., 2025) by analysing a larger MSR cohort (n = 44), reusing the same 10-child TAU comparison cohort, applying participant-level pre-post controlled analyses, and reporting multiplicity-adjusted interaction statistics. This sample overlap and the distinct contribution of the present analysis are disclosed explicitly.

Future research should employ randomized controlled designs, larger samples, standardized fidelity measures, and longitudinal follow-up to clarify mechanisms of change and evaluate the durability of observed effects. Further work should also investigate which clinical profiles are most responsive to sensory–motor interventions.

## Data Availability

The raw data supporting the conclusions of this article will be made available by the authors, without undue reservation.
