# Supplementary material for: Selective change in personal autonomy associated with motor sensory room training in children with autism: an exploratory controlled pre-post study
Source: Front Psychol. 2026 Oct 19;17:1933418. doi: 10.3389/fpsyg.2026.1933418 (PMC13724440; doi:10.3389/fpsyg.2026.1933418)
Supplement: Supplementary file 1 [file Supplementary_file_1.DOCX]

**Supplementary Material S1**

**Study-Specific Interview, Observational Items, and Coding Manual**

*A Motor Sensory Room Training for Children with Autism: A Controlled Pre–Post Study*

# 1. Status and intended use

This supplement documents the study-specific parent-interview and observational variables used in the Motor Sensory Room study. It was reconstructed from the item labels and scoring structure retained in the source dataset and from the operational procedure confirmed by the authors. It is not a newly validated instrument and must not be presented as a PEP-3 or Sensory Profile-2 scale. The PEP-3 was administered and interpreted according to its published manual; proprietary PEP-3 test items are not reproduced here.

The scale recorded the magnitude, consistency, independence, or functional impact of the behavior named by each item. For positively framed ability items, higher raw values represented more consistent or independent performance. For difficulty or dependence items, higher raw values represented greater difficulty, frequency, intensity, or support need. Difficulty items were reverse-scored only for construction of behavioral composites so that higher composite values indicated better functioning.

# 2. Assessment and consensus-coding procedure

Information was obtained from the parent interview and direct clinical observation at T1 and T2. Two observers were present during assessments. A category was entered into the study record only when both observers agreed. The authors confirmed that agreement was reached for every recorded rating. Only one agreed score per item and time point was retained; separate observer ratings were not archived. Thus, the observed procedure achieved complete consensus at data entry, but a chance-corrected coefficient such as weighted kappa or ICC cannot be reconstructed from the available database. Future applications should preserve the two independent ratings before consensus and double-code at least 20% of assessments.

# 3. General four-category rules

| **Score** | **Label** | **Ability/independence item** | **Difficulty, dependence, or response-magnitude item** |
| --- | --- | --- | --- |
| 0 | None | Behavior/ability not demonstrated, even with ordinary prompting. | Named behavior or observable response absent during the assessment/reference period. |
| 1 | Low | Emerging or occasional performance; substantial prompting or assistance required. | Mild, brief, or occasional behavior/response with little functional interference. |
| 2 | Medium | Functional but inconsistent performance; some prompting, supervision, or assistance required. | Clear or recurrent behavior/response with moderate interference or support need. |
| 3 | High | Consistent, generalized, and independent performance in the assessed context. | Frequent, intense, persistent, or prolonged behavior/response with substantial interference or support need. |

# 4. Behavioral interview items and domain anchors

## Feeding

| **Dataset item (English rendering)** | **Raw-score meaning** |
| --- | --- |
| Food selectivity | Difficulty |
| Preference for solid foods | Preference magnitude |
| Preference for liquid foods | Preference magnitude |
| Drinks independently | Ability/independence |

| **0 — None** | **1 — Low** | **2 — Medium** | **3 — High** |
| --- | --- | --- | --- |
| No relevant behavior/ability observed | Mild or occasional pattern; minimal interference / emerging independence | Recurrent pattern or partial independence; moderate support/interference | Persistent marked pattern or consistent independence, according to item direction |

Coding note: apply the general frequency, consistency, independence, support-need, and functional-impact rules in Section 3. Where the item is positively framed, higher values indicate greater ability. Where it is framed as a difficulty, dependence, preference, or behavior frequency, higher values indicate greater magnitude of that named feature.

## Sleep

| **Dataset item (English rendering)** | **Raw-score meaning** |
| --- | --- |
| Difficulty falling asleep | Difficulty |
| Light sleep | Difficulty |
| Sleeps independently | Ability/independence |
| Uses transitional objects to fall asleep | Dependence/support need |

| **0 — None** | **1 — Low** | **2 — Medium** | **3 — High** |
| --- | --- | --- | --- |
| Named sleep behavior absent / independence absent | Occasional mild difficulty or emerging independent sleep | Recurrent difficulty or partial independence with support | Persistent marked difficulty/dependence or consistent independent sleep |

Coding note: apply the general frequency, consistency, independence, support-need, and functional-impact rules in Section 3. Where the item is positively framed, higher values indicate greater ability. Where it is framed as a difficulty, dependence, preference, or behavior frequency, higher values indicate greater magnitude of that named feature.

## Motor coordination

| **Dataset item (English rendering)** | **Raw-score meaning** |
| --- | --- |
| Walks easily | Ability |
| Unsteady walking | Difficulty |
| Collides with obstacles | Difficulty |
| Loses balance | Difficulty |
| Toe walking | Behavior frequency |
| Tendency to run | Behavior frequency |

| **0 — None** | **1 — Low** | **2 — Medium** | **3 — High** |
| --- | --- | --- | --- |
| Named behavior/skill absent | Occasional or emerging performance; mild effect | Recurrent/functional but inconsistent performance; moderate effect or support | Frequent marked difficulty/behavior or consistently proficient performance |

Coding note: apply the general frequency, consistency, independence, support-need, and functional-impact rules in Section 3. Where the item is positively framed, higher values indicate greater ability. Where it is framed as a difficulty, dependence, preference, or behavior frequency, higher values indicate greater magnitude of that named feature.

## Play

| **Dataset item (English rendering)** | **Raw-score meaning** |
| --- | --- |
| Tends to isolate and play alone | Difficulty/behavior frequency |
| Plays with others | Ability |
| Restricted or absorbing interests | Difficulty/intensity |
| Functional use of toys | Ability |
| Interest in videogames and television | Engagement/frequency; not intrinsically positive or negative |

| **0 — None** | **1 — Low** | **2 — Medium** | **3 — High** |
| --- | --- | --- | --- |
| Named play behavior absent | Occasional/emerging engagement or mild difficulty | Recurrent/functional but inconsistent engagement or moderate difficulty | Consistent functional engagement or persistent/restrictive difficulty, by item direction |

Coding note: apply the general frequency, consistency, independence, support-need, and functional-impact rules in Section 3. Where the item is positively framed, higher values indicate greater ability. Where it is framed as a difficulty, dependence, preference, or behavior frequency, higher values indicate greater magnitude of that named feature.

## Personal autonomy

| **Dataset item (English rendering)** | **Raw-score meaning** |
| --- | --- |
| Dresses independently | Ability/independence |
| Washes independently | Ability/independence |
| Requires adult prompting to be autonomous | Dependence/support need |

| **0 — None** | **1 — Low** | **2 — Medium** | **3 — High** |
| --- | --- | --- | --- |
| Autonomous act absent / adult prompting absent | Emerging independence or occasional prompting | Partial independence or recurrent prompting | Consistent independence or persistent adult prompting, by item direction |

Coding note: apply the general frequency, consistency, independence, support-need, and functional-impact rules in Section 3. Where the item is positively framed, higher values indicate greater ability. Where it is framed as a difficulty, dependence, preference, or behavior frequency, higher values indicate greater magnitude of that named feature.

## Communication

| **Dataset item (English rendering)** | **Raw-score meaning** |
| --- | --- |
| Communicates needs | Ability |
| Uses verbal language | Ability/frequency |
| Uses non-verbal language | Ability/frequency |
| Tendency to remain silent | Difficulty/behavior frequency |

| **0 — None** | **1 — Low** | **2 — Medium** | **3 — High** |
| --- | --- | --- | --- |
| Named communication behavior absent | Emerging/occasional communication or silence | Functional but inconsistent communication or recurrent silence | Consistent communication or persistent silence, by item direction |

Coding note: apply the general frequency, consistency, independence, support-need, and functional-impact rules in Section 3. Where the item is positively framed, higher values indicate greater ability. Where it is framed as a difficulty, dependence, preference, or behavior frequency, higher values indicate greater magnitude of that named feature.

## Social interaction

| **Dataset item (English rendering)** | **Raw-score meaning** |
| --- | --- |
| Appropriate interaction with peers | Ability |
| Appropriate interaction with adults | Ability |
| Atypical social behavior with unfamiliar people | Difficulty |
| Tendency to social isolation | Difficulty/behavior frequency |

| **0 — None** | **1 — Low** | **2 — Medium** | **3 — High** |
| --- | --- | --- | --- |
| Named social behavior absent | Emerging/occasional appropriateness or mild atypicality | Functional but inconsistent interaction or recurrent atypicality | Consistently appropriate interaction or persistent marked atypicality/isolation |

Coding note: apply the general frequency, consistency, independence, support-need, and functional-impact rules in Section 3. Where the item is positively framed, higher values indicate greater ability. Where it is framed as a difficulty, dependence, preference, or behavior frequency, higher values indicate greater magnitude of that named feature.

## Behavioral regulation

| **Dataset item (English rendering)** | **Raw-score meaning** |
| --- | --- |
| Aggressive behavior toward others | Difficulty/frequency |
| Self-injurious behavior | Difficulty/frequency |
| Lack of interaction with others | Difficulty/frequency |
| Follows adult instructions | Ability |
| Oppositional behavior | Difficulty/frequency |

| **0 — None** | **1 — Low** | **2 — Medium** | **3 — High** |
| --- | --- | --- | --- |
| Named behavior absent / compliance absent | Occasional mild problem or emerging compliance | Recurrent problem with moderate impact or inconsistent compliance | Persistent severe problem or consistent compliance, by item direction |

Coding note: apply the general frequency, consistency, independence, support-need, and functional-impact rules in Section 3. Where the item is positively framed, higher values indicate greater ability. Where it is framed as a difficulty, dependence, preference, or behavior frequency, higher values indicate greater magnitude of that named feature.

# 5. Sensory-stimulus observation items and anchors

| **Modality** | **Stimulus item** |
| --- | --- |
| Auditory | Long sound |
| Auditory | Short sound |
| Auditory | Loud sound |
| Visual | Lights |
| Visual | Video projection |
| Gustatory | Solid food |
| Gustatory | Liquid food |
| Tactile | Rough surfaces |
| Tactile | Smooth surfaces |
| Tactile | Soft surfaces |
| Tactile/thermal | Cold sensations |
| Tactile/thermal | Warm sensations |
| Olfactory | Strong odors / withdrawal from strong odors |

| **Score** | **Operational sensory-response anchor** |
| --- | --- |
| 0 — None | No observable orienting, avoidance, seeking, affective, motor, or regulatory response to the presented stimulus. |
| 1 — Low | Subtle, brief, or inconsistent response; no meaningful disruption and little or no support required. |
| 2 — Medium | Clear and reproducible response of moderate intensity or duration; some change in engagement/regulation or limited support required. |
| 3 — High | Immediate, intense, persistent, or prolonged response with marked avoidance/seeking, dysregulation, interruption of the task, or substantial support required. |

Interpretation rule: these raw sensory categories quantify response magnitude, not clinical desirability. A high score may reflect hyper-reactivity, strong seeking, or another intense response, whereas a low score may reflect hypo-reactivity or tolerance. Improvement must therefore be judged against the child’s baseline response pattern and therapeutic target; numerical increase or decrease alone is insufficient.

# 6. Composite construction and missing data

For behavioral composites, positively framed ability items retained their 0–3 coding. Difficulty and dependence items were directionally reversed before averaging (reversed score = 3 − raw score), so higher composite scores represented better functioning. Preference/frequency items should be included only when their clinical direction is prespecified for that domain. Domain scores are the arithmetic mean of available directionally aligned items. The number of observed items contributing to every domain score should be retained; a domain should not be calculated when more than 20% of its planned items are missing. Sensory items remain item-level raw scores and are not combined into an improvement score without a prespecified hypo-/hyper-reactivity classification.

# 7. Recommended reliability procedure for future studies

- Two trained observers should score the same assessment independently before discussing the case.
- Separate observer-level item ratings must be archived before consensus.
- At least 20% of assessments should be randomly selected for double coding across both time points and study groups.
- Weighted Cohen’s kappa should be reported for ordinal item ratings; an absolute-agreement ICC may additionally be reported for domain composites.
- The weighting scheme, ICC model, confidence intervals, number of double-coded assessments, and handling of missing ratings should be specified.
- Consensus ratings may be used for the analysis dataset only after independent scores have been preserved.

# 8. Methodological note

This manual increases transparency but does not constitute a retrospective psychometric validation. Published reliability estimates for the PEP-3 concern standardized PEP-3 administration and cannot be transferred to the study-specific interview or sensory-response variables. The current study reports complete consensus between the two observers as confirmed by the authors, while explicitly acknowledging that the lack of separately archived ratings precludes verification and chance-corrected reliability estimation.

# References supporting the reporting approach

1. Schopler, E., Lansing, M. D., Reichler, R. J., & Marcus, L. M. (2005). Psychoeducational Profile, Third Edition (PEP-3). PRO-ED.
2. Siper, P. M., et al. (2021). A clinician-administered observation and corresponding caregiver interview capturing DSM-5 sensory reactivity symptoms in children with ASD. Autism Research. https://pmc.ncbi.nlm.nih.gov/articles/PMC8524392/
3. Mulligan, S., et al. (2023). An observed assessment of sensory responsivity in autism spectrum disorders: Associations with diagnosis, age, and parent report. https://pmc.ncbi.nlm.nih.gov/articles/PMC9898461/
4. Savarese, G., et al. (2025). Preliminary results of Sensorimotor Room training for the improvement of sensory and motor skills in children with autism spectrum disorders. Pediatric Reports, 17(1), 4. https://doi.org/10.3390/pediatric17010004
